# Severe Wound by a Circular Saw

**Published:** 1851-12

**Authors:** Wm. J. Henderson

**Affiliations:** Lock-Haven, Pennsylvania


*Severe Wound by a Circular Saw.* By Wm. J. Henderson, M.D. Lock-Haven, Pennsylvania.

On the seventh of May last, I was called to Farransville, six miles from home, to see G—— S——, who, having fallen asleep in the mill, was caught by a circular saw which inflicted a severe and extensive wound in the back. I found the patient lying on the floor, weltering in blood from the wound, which had been hastily sewed together, until I should arrive. The wound was parallel with the spine, and about one and a half inches from that column. On measuring I found the following dimensions: length 8 inches, 6 wide, and one and a half deep. The contractions of the abdominal muscles caused the wound to gap to such an extent, that I laid both my hands flat between the edges, which were ragged and contused; the muscles of the spine were torn and came away in shreds. The ninth and tenth ribs were completely divided about an inch from the articulations, together with their accompanying arteries, which bled very little. Portions of the shirt and vest were sticking in the wound, and much clotted blood.

After cleaning the wound of all foreign matters, and clipping off the ragged edges, it was closed with five sutures and adhesive straps; the cold water dressing applied, and the Pulv. Doveri prescribed. Next day the patient said he felt quite well, had slept soundly all night, and had no pain in the wound. He continued rapidly to convalesce without any bad symptoms, and on the sixth day walked down stairs and rode in a wagon four miles; and in two weeks from the injury he paid me a visit in town having walked all the way, perfectly restored; the wound having closed without any secretion of pus, and the divided ribs having firmly united.

Fortunately for the patient, the bench on which the saw ran was lower on one side, and being drawn down on his back, the saw cut *obliquely* towards the spine. Four lines deeper the cavities of the chest and abdomen would have been penetrated.

